# Leveraging 3D convolutional neural network and 3D visible-near-infrared multimodal imaging for enhanced contactless oximetry

**DOI:** 10.1117/1.JBO.29.S3.S33309

**Published:** 2024-08-21

**Authors:** Wang Liao, Chen Zhang, Belmin Alić, Alina Wildenauer, Sarah Dietz-Terjung, Jose Guillermo Ortiz Sucre, Sivagurunathan Sutharsan, Christoph Schöbel, Karsten Seidl, Gunther Notni

**Affiliations:** a Ilmenau University of Technology, Department of Mechanical Engineering, Ilmenau, Germany; b University of Duisburg-Essen, Chair of Electronic Components and Circuits, Duisburg, Germany; c University Medicine Essen, Ruhrlandklinik, Chair of Sleep and Telemedicine, Essen, Germany; d University Medicine Essen, Ruhrlandklinik, Department of Pneumology, Essen, Germany; e Fraunhofer Institute for Applied Optics and Precision Engineering, Jena, Germany

**Keywords:** oxygen saturation, contactless oximetry, multimodal imaging, deep learning

## Abstract

**Significance:**

Monitoring oxygen saturation (${\mathrm{SpO}}_{2}$) is important in healthcare, especially for diagnosing and managing pulmonary diseases. Non-contact approaches broaden the potential applications of ${\mathrm{SpO}}_{2}$ measurement by better hygiene, comfort, and capability for long-term monitoring. However, existing studies often encounter challenges such as lower signal-to-noise ratios and stringent environmental conditions.

**Aim:**

We aim to develop and validate a contactless ${\mathrm{SpO}}_{2}$ measurement approach using 3D convolutional neural networks (3D CNN) and 3D visible-near-infrared (VIS-NIR) multimodal imaging, to offer a convenient, accurate, and robust alternative for ${\mathrm{SpO}}_{2}$ monitoring.

**Approach:**

We propose an approach that utilizes a 3D VIS-NIR multimodal camera system to capture facial videos, in which ${\mathrm{SpO}}_{2}$ is estimated through 3D CNN by simultaneously extracting spatial and temporal features. Our approach includes registration of multimodal images, tracking of the 3D region of interest, spatial and temporal preprocessing, and 3D CNN-based feature extraction and ${\mathrm{SpO}}_{2}$ regression.

**Results:**

In a breath-holding experiment involving 23 healthy participants, we obtained multimodal video data with reference ${\mathrm{SpO}}_{2}$ values ranging from 80% to 99% measured by pulse oximeter on the fingertip. The approach achieved a mean absolute error (MAE) of 2.31% and a Pearson correlation coefficient of 0.64 in the experiment, demonstrating good agreement with traditional pulse oximetry. The discrepancy of estimated ${\mathrm{SpO}}_{2}$ values was within 3% of the reference ${\mathrm{SpO}}_{2}$ for ∼80% of all 1-s time points. Besides, in clinical trials involving patients with sleep apnea syndrome, our approach demonstrated robust performance, with an MAE of less than 2% in ${\mathrm{SpO}}_{2}$ estimations compared to gold-standard polysomnography.

**Conclusions:**

The proposed approach offers a promising alternative for non-contact oxygen saturation measurement with good sensitivity to desaturation, showing potential for applications in clinical settings.

## 1 Introduction

Vital signs, such as body temperature, heart rate, respiratory rate, and blood pressure are standard indicators of an individual’s physiological functions in most medical settings.^1^ Monitoring these vital parameters is crucial for early diagnosis, medical treatment, risk assessment, and patient recovery monitoring.^2^^,^^3^ With the advancement of medical measurement technology, oxygen saturation has increasingly become recognized as an indispensable fifth vital sign.^4^ Oxygen saturation indicates the percentage of oxygenated hemoglobin (${\mathrm{HbO}}_{2}$) and hemoglobin (Hb) in the blood, in which the artery should be in the range of 95% to 100% in healthy individuals.^5^ Many pulmonary diseases cause abnormalities in oxygen saturation values, such as acute pneumonia, chronic obstructive pulmonary disease (COPD), and sleep apnea syndrome (SAS). Furthermore, the outbreak of coronavirus (COVID-19) has further underscored the critical importance of oxygen saturation measurement.

The gold standard for measuring arterial oxygen saturation (${\mathrm{SaO}}_{2}$) is the invasive arterial blood gas (ABG) test,^6^ which is performed by medical professionals. Mixed venous oxygen saturation (${\mathrm{SvO}}_{2}$) is normally measured via a pulmonary artery catheter. Non-invasive methods based on near-infrared spectroscopy are developed to measure tissue oxygen saturation (${\mathrm{StO}}_{2}$), which directly provides an assessment of the oxygenation status of tissues. Time-domain near-infrared spectroscopy (TD-NIRS) is an established technique, which allows the estimation of ${\mathrm{StO}}_{2}$ at multiple depths, including beyond 2 cm deep.^7^^,^^8^ This capability opens a range of applications, such as determining ${\mathrm{StO}}_{2}$ in the brain.^9^ The estimation of ${\mathrm{SaO}}_{2}$ at peripheral capillary is called ${\mathrm{SpO}}_{2}$. A non-invasive pulse oximeter is known for its convenience for real-time ${\mathrm{SpO}}_{2}$ estimation. Polysomnography (PSG) systems ^10^ used in sleep monitoring also incorporate pulse oximeter to record ${\mathrm{SpO}}_{2}$ overnight. A typical pulse oximeter employs a light source that projects red and infrared light onto fingertips or earlobes. Oxygenated hemoglobin and deoxygenated hemoglobin exhibit distinct characteristics of absorption spectra. By contrasting the transmitted light intensities at 660 and 940 nm wavelengths captured by the photoelectric sensor, the pulse oximeter determines the ${\mathrm{SpO}}_{2}$ by utilizing the ratio-of-ratios (RR) method.^11^ However, contact-based methods face challenges for patients with infectious diseases or allergies,^12^ especially during long-term measurements such as sleep monitoring. To overcome these limitations of contact-based methods, there is an increasing focus on camera-based ${\mathrm{SpO}}_{2}$ measurement. Bui et al.^13^ and Ding et al.^14^ utilized a camera-based approach, where participants placed a finger over the smartphone’s camera and flash, diverging from true contactless methods. Many studies on contactless ${\mathrm{SpO}}_{2}$ measurements usually use red, green, and blue (RGB) cameras to capture hands^15^^,^^16^ or faces^17^^–^^19^ with ambient light and extract weak pulsatile temporal features from remote photoplethysmogram (rPPG) signals through different analytical filtering techniques^20^^–^^22^ or neural networks^23^^–^^25^ to calculate ${\mathrm{SpO}}_{2}$. Acquiring high-quality rPPG signals is a challenging task, which can be affected by factors like illumination conditions, sampling rate, and sensor noise, along with disruptions from facial movements such as smiles or blinks, which compromise ${\mathrm{SpO}}_{2}$-related information. The spatial encoded patterns of the captured skin regions have been proven by Wieringa et al.^26^ and Rosa and Betini^27^ to contain oxygen saturation information. Hu et al.^28^ employed a 2D residual cascade and coordinate attention mechanism to analyze feature channel correlations of spatial data, using neural networks to extract and concatenate spatial features for estimation. Few studies simultaneously consider both spatial and temporal features. To fill in the gap, in our previous work,^29^ 3D convolutional network (3D CNN) are used to extract spatial-temporal information from the near-infrared multispectral videos for ${\mathrm{SpO}}_{2}$ estimation. Besides, in our literature review scope, we observed that current research gaps of camera-based contactless ${\mathrm{SpO}}_{2}$ measurement include region of interest (ROI) tracking, acquiring datasets with significant ${\mathrm{SpO}}_{2}$ fluctuations, and validation in clinical settings. We noted that most studies are based on datasets containing only a few instances of low ${\mathrm{SpO}}_{2}$ levels and the overwhelming majority of ${\mathrm{SpO}}_{2}$ ranges between 95% and 100%. To address these challenges, in this work, we propose a 3D convolutional neural networks-based approach to estimate ${\mathrm{SpO}}_{2}$ from videos captured by our 3D visible-near-infrared (VIS-NIR) multimodal camera system. The performance is verified through both short-term daytime measurements on healthy participants and continuous long-term nighttime monitoring of patients with sleep apnea. The contributions of this work include the following:

1. We utilized a 3D VIS-NIR multimodal camera system to capture multimodal facial videos and proposed steps including multimodal image registration, 3D ROI tracking, spatial and temporal preprocessing, and 3D CNN-based spatial-temporal features extraction to enable oxygen saturation estimation in both during day and night.
2. We conducted a breath-holding study on 23 healthy participants with different skin types, achieving an MAE of 2.31 and a Pearson correlation coefficient of 0.64 compared to the reference oxygen saturation ranging from 80% to 99% measured by pulsed oximeter on the fingertip. In addition, our approach was also validated by a trial study involving long-term overnight monitoring of four real sleep disorder patients, demonstrating good agreement with the gold standard PSG.
3. We discussed various feature extraction strategies, different image channel combinations, and diverse neural network architectures (including light-weight networks) for their capability and performance to estimate ${\mathrm{SpO}}_{2}$ from 3D VIS-NIR multimodal videos.

## 2 Proposed Approach Based on Multimodal Imaging

Multimodal imaging refers to the integration of various imaging modalities such as 3D imaging, multispectral imaging, and thermal imaging. It allows for enhanced and more dependable analysis to realize intricate tasks^30^^–^^33^ based on diverse feature combinations from different imaging modalities. In our work, we use four imaging modalities, which include images from color (RGB) cameras, NIR 780 and NIR 940 nm cameras, and disparity maps produced by active stereo matching based on two NIR 850 nm cameras and GOBO projector.^34^ The details of our camera system setup will be introduced in Sec. 3. In this section, the proposed approach will be introduced, detailing how to regress ${\mathrm{SpO}}_{2}$ using 3D CNN from multimodal video sequences after multimodal image registration, 3D ROI tracking, and spatial and temporal preprocessing.

### 2.1 Multimodal Image Registration

**Fig. 1:**
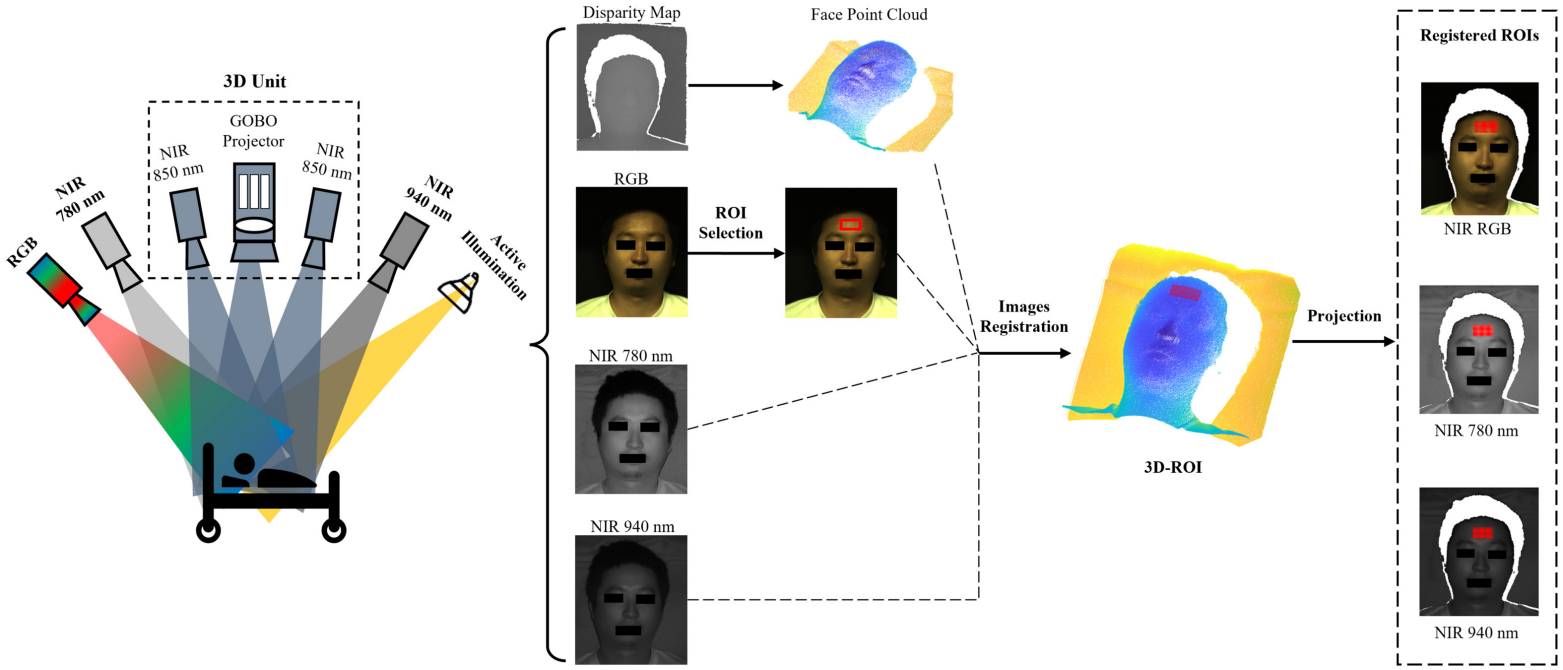
Schematic of 3D information-based multimodal facial image registration.

For the purpose of pixel-wise fusion of information from different 2D modalities, the 2D images were registered together using 3D information. Camera calibration is always the initial step. The intrinsic parameters of the two NIR cameras for stereo matching and also other 2D cameras are calibrated using Zhang’s algorithm.^35^ Simultaneously, the extrinsic camera parameters are calculated with respect to a reference 2D camera, for example, the RGB camera, using the method introduced in Ref. 36. Based on the NIR 850 nm camera parameters, a disparity map can be converted to a 3D point cloud. Assume $\left(u_{i},v_{i}\right)$ is the projection of one 3D point $\left(x_{i},y_{i},z_{i}\right)$ of the point cloud on the image plane of one of the 2D cameras (RGB, NIR 780 nm or NIR 940 nm), the transformation can be calculated as follows:

$$s\cdot [\begin{array}{c} u_{i}\\ v_{i}\\ 1 \end{array}]=K_{c}\cdot (R_{c}\cdot R_{\mathrm{rect}}^{-1}\cdot [\begin{array}{c} x_{i}\\ y_{i}\\ z_{i} \end{array}]+T_{c}),$$ (1)

where s is a factor for the normalization of homogeneous 2D points, $K_{c}$ is the intrinsic parameters matrix of this camera, $R_{c}$ and $T_{c}$ are the rotation matrix and translation vector of this camera, and $R_{\mathrm{rect}}$ is the rotation matrix of the reference camera for stereo rectification. When the projected image point does not align precisely with a pixel, bilinear interpolation among adjacent pixels is performed. Through this method, each 2D image captured by the cameras can be accurately mapped to the corresponding 3D point cloud. In this way, once an ROI is selected on a 2D image modality, it can be converted to the corresponding 3D ROI. The 3D ROI can be projected onto the images from other 2D cameras to assign gray values to these 3D points. In our work, the forehead region was used as the ROI for ${\mathrm{SpO}}_{2}$ estimation because of good blood flow, thin epidermis, and no hair.^37^^,^^38^ As shown in Fig. 1, we select a forehead region with width h and height w as ROI (h,w,3) on the color face image, and it can be converted to a 3D ROI. This 3D ROI is then projected to NIR 780 and NIR 940 nm images to obtain registered NIR 780 nm ROI (h,w,1) and NIR 940 nm ROI (h,w,1), from which corresponding gray values can be obtained.

### 2.2 Face Analysis and 3D ROI Tracking

**Fig. 2:**
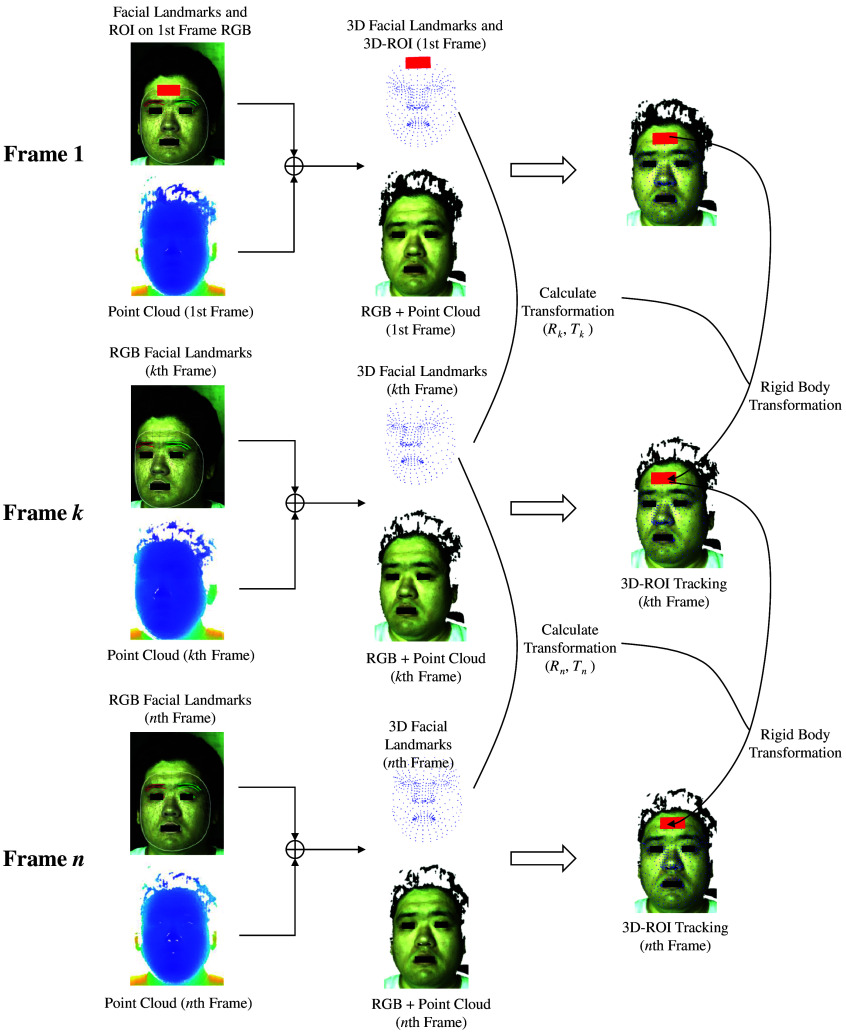
Illustrative example of the 3D ROI tracking across sequential frames.

For a continuous, registered multimodal facial video, we firstly utilized the MediaPipe Face Mesh framework,^39^ a pretrained, light-weight deep learning model, for high-precision facial feature extraction, leveraging its capability to identify and track 468 distinct landmarks across various facial regions on RGB video. Each landmark, along with its image coordinates, is uniquely indexed, enabling us to perform automatic video anonymization. This is achieved by pinpointing the landmarks of the eyes and mouth regions in each frame and overlaying black rectangles over these areas across all registered imaging modalities. Subsequently, the image coordinates of landmarks on the forehead region in the first frame of the RGB video are used to define a forehead 2D ROI, which is then converted to 3D ROI. From the second frame onwards, the 3D ROI was tracked based on the 2D coordinates of the facial landmarks and the 3D point cloud as shown in Fig. 2. At each frame, the facial landmarks will be converted to the registered point cloud as 3D facial landmarks. Let the set of 3D facial landmarks on the first video frame be denoted as $P_{1}=\{p_{1i}\in R^{3}|i=1,2,\ldots ,n\}$, where each $p_{1i}$ is a 3D point represented as a column vector in homogeneous coordinates $p_{1i}=[x_{1i},y_{1i},z_{1i},1{]}^{T}$. Similarly, for the kth frame, the set of corresponding 3D facial landmarks is $P_{k}=\{p_{ki}\in R^{3}|i=1,2,\ldots ,n\}$, with each landmark $p_{ki}$ also represented in homogeneous coordinates $p_{ki}=[x_{ki},y_{ki},z_{ki},1{]}^{T}$. Assume that the head is a rigid body, which means that the participant’s facial expression was unchanged over the video period. To model the current 3D head pose relating to the 3D face pose on the first frame, the rigid body transformation with six degrees of freedom (DoF) from $P_{1}$ to $P_{k}$ described by a rotation $R_{k}$ and a translation $t_{k}$ can be estimated as follows:

$$(R_{k},t_{k})=\underset{R,t}{\arg\;\min}\;\overset{n}{\underset{i=1}{\sum }}{\left\|Rp_{1i}+t-p_{ki}\right\|}^{2}.$$ (2)

**Fig. 3:**
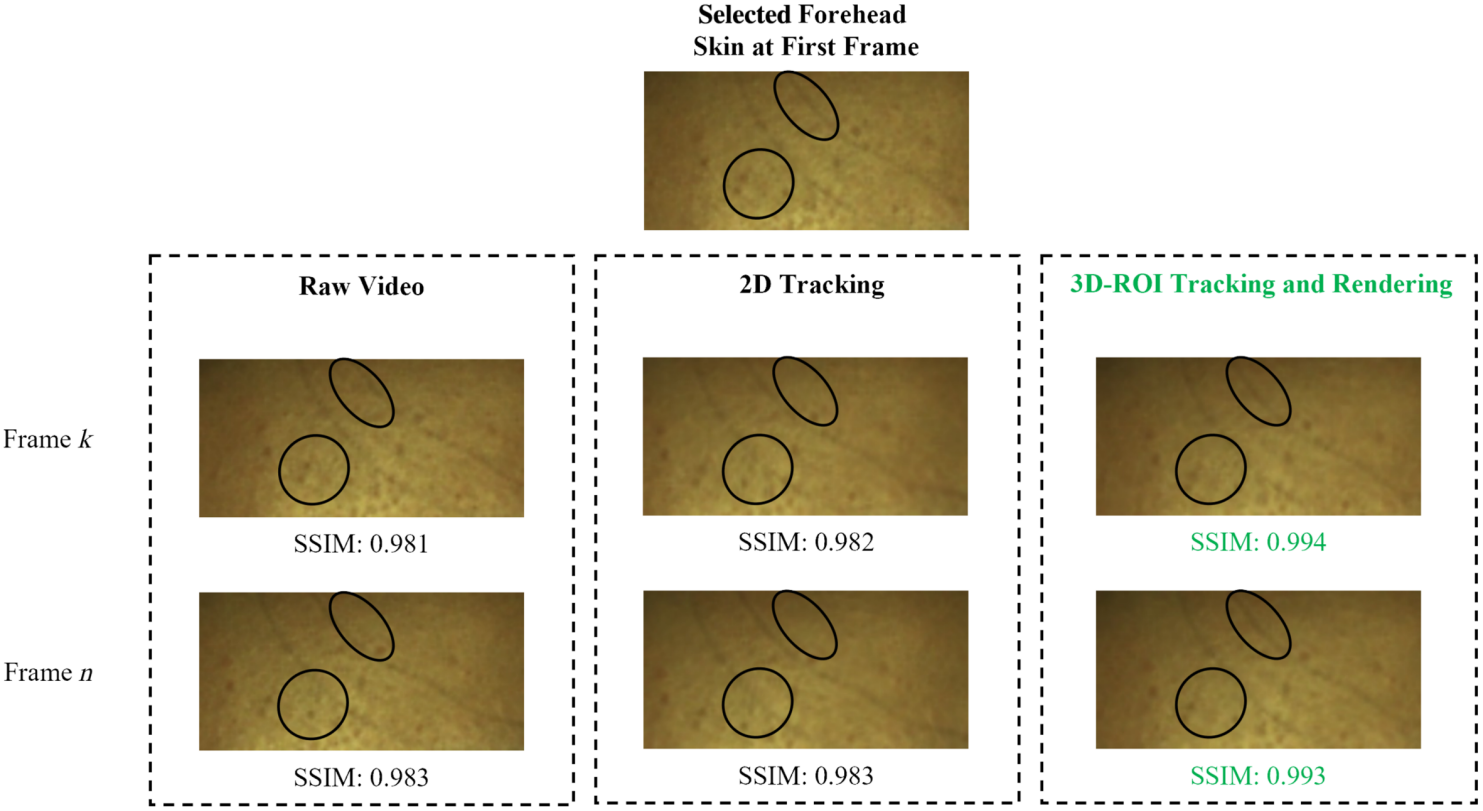
Comparative analysis of ROI tracking for a forehead region initialized in the first frame of a video sequence. The black elliptical outlines in the ROI highlight reference features such as hair and skin hyperpigmentation, serving as markers to intuitively observe the tracking performance. Structural similarity (SSIM) is calculated to quantitatively assess the tracking performance.

Thus, by employing the rotation $R_{k}$ and the translation $t_{k}$, all points within the 3D ROI defined on the first frame can be transformed to their corresponding positions on the kth frame. Head movements typically occur in three dimensions, not confined to a single plane. Tracking a fixed skin area is evidently more suitable using 3D information, whether there is significant movement or subtle involuntary motion. As shown in Fig. 3, we demonstrate the tracking effectiveness when projecting the tracked 3D ROI back into an RGB 2D ROI. One of the participants is instructed to remain as still as possible for 4 min. However, slight involuntary head movements are inevitable. Whether assessing reference regions visually or evaluating by structural similarity (SSIM), the proposed 3D-based tracking method can more exactly track the ROI throughout the video.

### 2.3 Spatial and Temporal Preprocessing

**Fig. 4:**
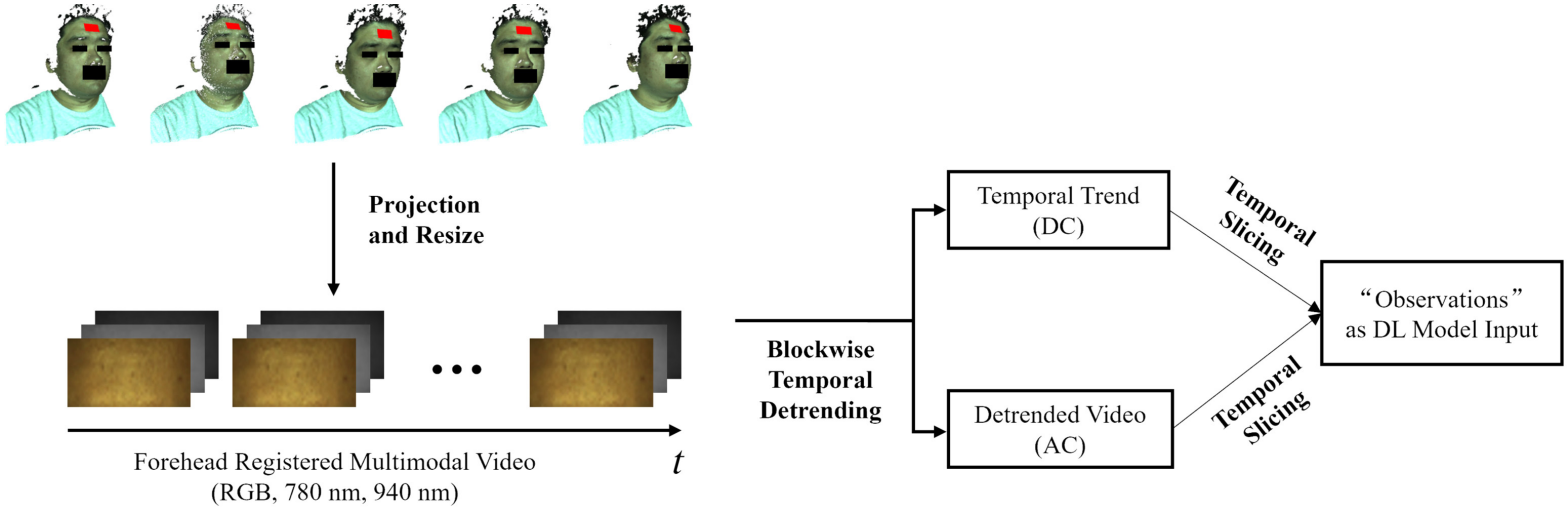
Process flow from 3D ROIs of a video sequence to input of the deep learning model.

As shown in Fig. 4, the tracked 3D ROI of the head in a video can be projected onto each modality to obtain 2D ROI videos. When these modalities are concatenated, a registered multimodal forehead ROI video is formed, encompassing five channels including R, G, B, 780, and 980 nm. Then, spatial and temporal preprocessing is applied. Assuming there is a multimodal forehead ROI video V, and for a given channel, each frame has a height h and width w. The videos are spatially partitioned into m×n block videos, with each block video spatially sized $\left\lfloor \frac{h}{m}\rfloor \times \lfloor \frac{w}{n}\right\rfloor$, discarding residual pixels at the edges. For the ith block video in a specific channel, each of its pixel values can be represented as $B_{i}(x,y,t)$, where x and y denote spatial coordinates and t denotes time. A cubic polynomial $P_{i}=a_{i}t^{3}+b_{i}t^{2}+c_{i}t+d_{i}$ can be fitted as the temporal trend of $B_{i}$:

$$(a_{i},b_{i},c_{i},d_{i})=\underset{a_{i},b_{i},c_{i},d_{i}}{\arg\;\min}\;\underset{t}{\sum }{\left(\frac{1}{\left|B_{i}\right|}\underset{x,y\in B_{i}}{\sum }B_{i}(x,y,t)-P_{j}\right)}^{2}.$$ (3)

Thus, for a certain pixel value of this $B_{i}$, it can be decomposed into the trend part $T_{i}(x,y,t)=P_{i}(t)$ and the detrended part $B_{i}^{\prime }(x,y,t)=B_{i}(x,y,t)-P_{i}(t)$. This blockwise temporal detrending is replicated across all blocks and five channels, decomposing the multimodal forehead ROI video V into two components: one devoid of temporal trend, presumably carrying more information similar to the AC component in traditional methods, and the trend component, encapsulating more DC component information. Then, these two parts of the video are temporally sliced into 15 frames detrended video sequences and trend video sequences of 1-s time length, respectively. Concatenating a detrended video sequence and a trend video sequence forms an “observation,” which serves as the input to the deep learning model.

### 2.4 Oxygen Saturation Regression with 3D CNN

These observations serve as input of spatial-temporal convolutional layers for feature extraction. Spatial-temporal convolution, also known as 3D convolution, enhances the feature extraction ability on volumetric data, thereby integrating information across various spatial dimensions and the temporal axis.^40^ The 3D convolutional kernel slides across the input “observation,” computing a dot product between its learnable weights and the corresponding local regions of the input at each position.

**Fig. 5:**
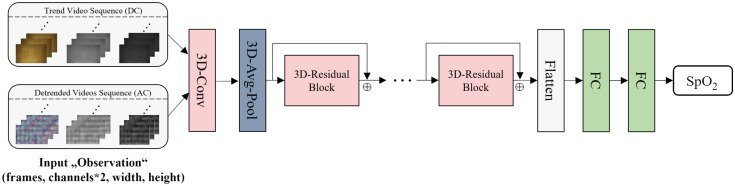
Neural network structure for oxygen saturation estimation.

As shown in Fig. 5, we use a ResNet 18^41^-like structure with 3D convolution as a feature extractor. The input “observation” is firstly fed into a 3D convolutional layer with a kernel size of [7,7,7] and then forwarded to four residual blocks with a convolutional kernel size of [3,3,3]. To accentuate global feature representation while diminishing the focus on local textural details, a 3D global average pooling layer is situated before the residual blocks. The extracted features are flattened to the feature vector as the input of the regressor, which is composed of two fully connected layers (FC). The output of the regressor is normalized to be between 0 and 1, which is estimated ${\mathrm{SpO}}_{2}$ after scaling. Every “observation” is associated with one ${\mathrm{SpO}}_{2}$ output from the regressor and a reference value. For training the neural network, mean square error (MSE) is used as the loss function and Adam^42^ is chosen as the optimizer. We use both dropout and early stopping to prevent overfitting. Hyperparameters are set empirically. Neither commercial pulse oximeters nor clinical devices used for oximetry analysis provide decimal values, so we obtained oxygen saturation reference values as integers. Although neural networks are capable of producing outputs with decimals, we have rounded the outputs as the only post-processing step.

## 3 Experiment Setting and Data Acquisition

### 3.1 Multimodal Imaging Camera System

**Fig. 6:**
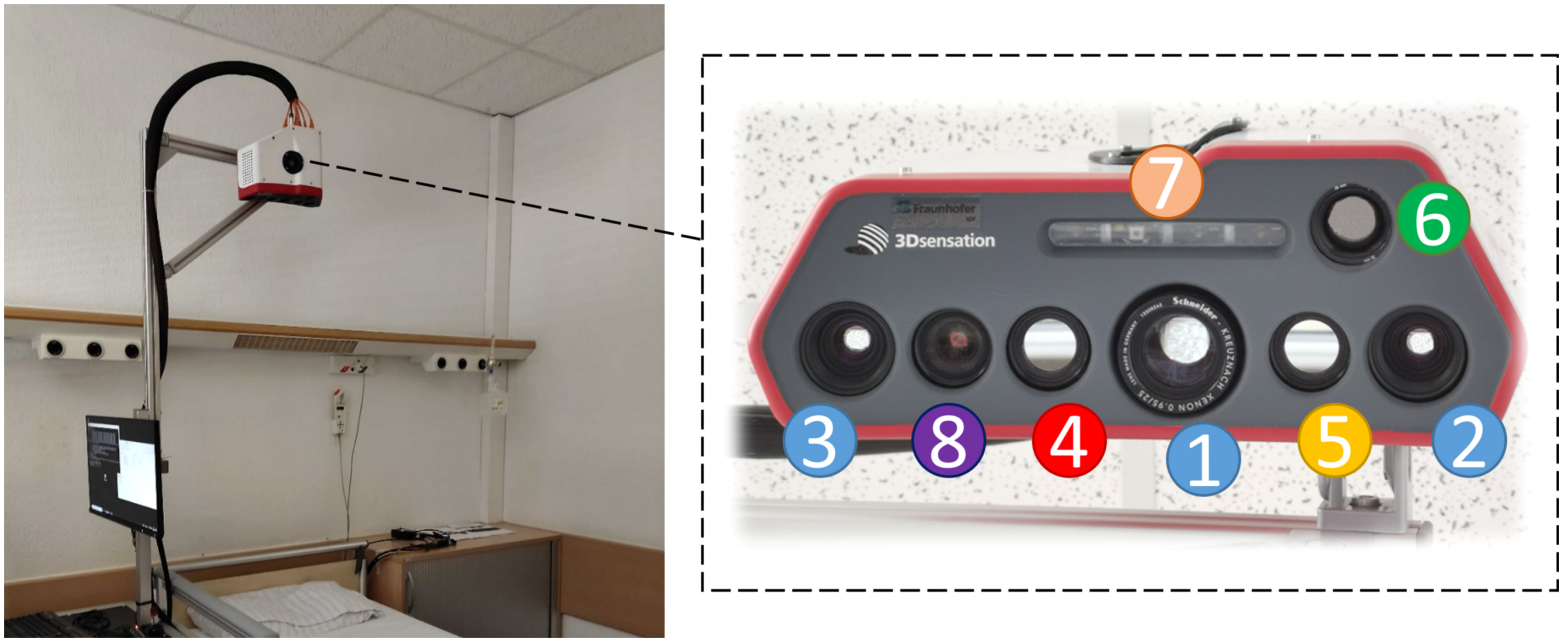
Multimodal camera system with sensor head composed of a GOBO projector (1), two NIR cameras at 850 nm (2, 3), NIR camera at 780 nm (4), NIR camera at 940 nm (5), thermal camera (6), LED array with LEDs at 780 and 940 nm (7), and color camera (8).

We utilized a multimodal imaging system manufactured by the Fraunhofer Institute for Applied Optics and Precision Engineering in our previous work^43^ and established an experimental setup at University Medicine Essen as shown in Fig. 6.

The sensor head of this camera system contains a real-time 3D sensor unit composed of two NIR 850 nm high-speed cameras with a full width of half maximum (FWHM) of 50 nm and a high-speed GOBO projector^34^ at the same light wavelength. Besides the 3D sensor, a color camera, two NIR cameras at 780 and 940 nm, and a thermal camera are integrated into the housing. In this study, the thermal camera is inactive, which is integrated for the estimation of other vital signs. The frame rates of these 2D cameras are 15 Hz, and they are hardware-triggered and synchronized with the 3D video stream. The spatial resolution of these active 2D cameras is 896×704. The system utilizes a light-emitting diode (LED) array for homogeneous illumination, comprising one LED operating at 780 nm and three LEDs at 940 nm. Each LED in the array has a beam angle within half-maximum intensity ranging from 90 deg to 120 deg, with an output power of 1 W. The camera system encompasses a lateral measurement field of ∼500  mm by 400 mm when positioned at an intermediate distance of 1.5 m, and the cumulative irradiation from this LED array configuration is $∼1.255\;\mu W/{\mathrm{mm}}^{2}$, thereby adhering to the safety standards for ocular exposure.^44^

### 3.2 Video Data Acquisition and Reference Value Recording

To validate our approach, a total of 23 cardiopulmonary healthy participants (numbered Par#1 to Par#23) were recruited for a breath-holding study. The study is approved by the Ethics Committee of the Faculty of Medicine, University of Duisburg-Essen (approval no. 21-10312-BO). Informed consent was obtained from all individual participants included in this experiment. Their Fitzpatrick skin types^45^ range from type II to type V. To obtain video data with low ${\mathrm{SpO}}_{2}$ values, participants were expected to exhale as much as possible and then hold their breath for a while during a video shoot. For comfort and health reasons, the duration of breath-holding was determined by the participants themselves. When they felt they could not tolerate breath-holding anymore, they would breathe normally for a period of time. Participants repeated the cycle of exhalation, breath-holding, inhalation, and normal breathing three times in ∼4  min. While we advised participants to face the camera system with the front view, we could not constrain their head movements. Especially, the breath-holding can lead to momentary discomfort, resulting in some unavoidable involuntary movements. Participants were engaged in two separate measurements, interspersed with a 5-min break to regulate their breathing. During the video capture, a Pulox PO-200 pulse oximeter was clipped to the fingertip to measure the participant’s reference ${\mathrm{SpO}}_{2}$ values. A webcam was used to capture the pulse oximeter display with a frame rate of 1 Hz. Pre-trained optical character recognition model by EasyOCR was used to read the ${\mathrm{SpO}}_{2}$ reference from captured displays. By holding breath, the ${\mathrm{SpO}}_{2}$ value can drop below 95%, which is considered the lower limit of the normal range,^46^ and some participants can even drop to 80%. After the participant resumes normal breathing, the ${\mathrm{SpO}}_{2}$ will quickly return to the healthy range. Captured video and reference recording were first synchronized by timestamps. It is worth mentioning that since the face and fingertips are different parts of the body, the synchronization in recording time does not mean that the ${\mathrm{SpO}}_{2}$ obtained from facial videos and those obtained from fingertip pulse oximeters are synchronized. According to Refs. 47 and 48, the ${\mathrm{SpO}}_{2}$ obtained from facial videos are ∼20  s. faster than those obtained from fingertip pulse oximeters. Therefore, in our subsequent experiments, for training, we applied a constant 20 s time advance to the reference values from the pulse oximeter. For evaluating the inference, we shifted the reference time trace within a range of 20  s±5  s to maximize its correlation with the estimated time trace.

**Fig. 7:**
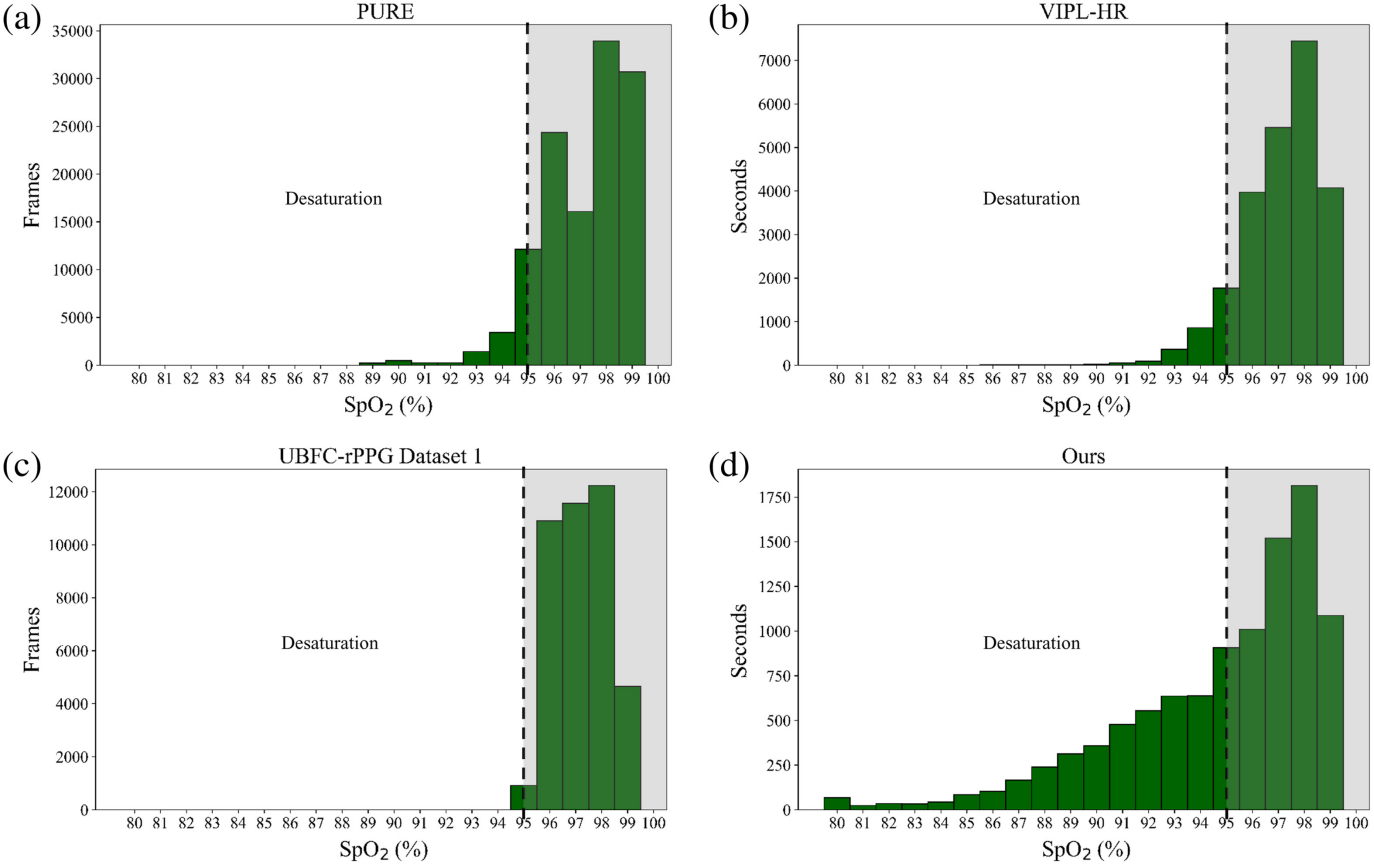
Histograms of reference ${\mathrm{SpO}}_{2}$ distributions in benchmark and our datasets. In PURE and UBFC-rPPG dataset 1, each sample corresponds to one frame, while in VIPL-HR and our dataset, each sample represents 1 s. The dashed line to the left indicates desaturation, that is, ${\mathrm{SpO}}_{2}$ values below 95%.

Through our experiment, a total of 168.5 min of multimodal videos were captured, equating to 10,112 1-s “observations” after preprocessing. Each 1-s “observation” corresponds to a specific ${\mathrm{SpO}}_{2}$ value. The reference ${\mathrm{SpO}}_{2}$ values ranged from 80% to 99%. In our literature review, we observed that open-source datasets for camera-based ${\mathrm{SpO}}_{2}$ estimation are scarce, and no studies utilizing 3D VIS-NIR multimodal imaging have been found. Most of the datasets do not focus on ${\mathrm{SpO}}_{2}$ but rather on heart rate and respiration. Within our research scope, we found the PURE,^49^ VIPL-HR,^50^ and UBFC-rPPG^51^ datasets. In the PURE dataset, the researchers used an RGB camera to record 10 healthy subjects. The VIPL-HR dataset includes 107 healthy participants, mostly recorded with RGB modality, and a few with both RGB and NIR multispectral videos. The UBFC-rPPG dataset has only a few participants with ${\mathrm{SpO}}_{2}$ reference values and includes only RGB videos. It can be seen from Table 1, that within the limited camera-based benchmark datasets available, reference ${\mathrm{SpO}}_{2}$ values rarely drop below the healthy range, with almost no instances falling below 90%. Despite the challenges associated with obtaining data on low ${\mathrm{SpO}}_{2}$ levels, our dataset successfully includes 40% of “observations” with desaturation. Specifically, it encompasses 2.82% of “observations” with ${\mathrm{SpO}}_{2}$ from 80 to 85 and 11.66% within the ${\mathrm{SpO}}_{2}$ range 86% to 90%. In addition, the 25th percentile (Q1) of ${\mathrm{SpO}}_{2}$ reference in our dataset is located at 92%. A comparison of four histograms has been shown in Fig. 7, representing the distribution of ${\mathrm{SpO}}_{2}$ values in different datasets. Unlike the other datasets, which show a steep decline in instances frequency below 95%, ours maintains a more gradual decrease, including many lower values. This suggests that our dataset captures a broader instances spectrum of ${\mathrm{SpO}}_{2}$ values, potentially offering richer insights for desaturation scenarios.

**Table 1:** Comparison of reference ${\mathrm{SpO}}_{2}$ coverage and distribution of benchmark datasets with ours.

Dataset	${\mathrm{SpO}}_{2}$ (%) Coverage				Centile of ${\mathrm{SpO}}_{2}$ (%)		
	[80, 85]	[86, 90]	[91, 95]	[96, 100]	5th percentile	Q1	Median
PURE^49^	-	0.55	14.19	85.26	95	96	98
VIPL-HR^50^	-	0.20	13.02	86.78	94	96	97
UBFC-rPPG^51^	-	-	2.27	97.73	96	96	97
Ours	2.82	11.66	31.78	53.74	87	92	96

## 4 Results and Discussion

As introduced in the previous section, this work involved 23 participants, each of whom was recorded in two separate around 4-min measurement sessions. We embarked on our validation by addressing a “participant-dependent” scenario, also referred to as “precision healthcare” validation, which means using one measurement from each participant as training data, with the subsequent measurement serving as the test data. This scenario emphasizes personalized analysis. Our focal point of result discussion shifts towards a more practical and generalizable scenario known as the “participant-independent” scenario or “leave-one-participant-out” validation. We systematically designate the two measurements of each participant as the test data while utilizing all available measurements from the remaining 22 participants as the training dataset. This strategy is aimed at validating the robustness and generalizability of our approach across different subjects. We will also explore the performance of various feature extraction strategies and the corresponding network architectures. In addition, we will also present test results and application scenarios with different input modalities. Finally, a clinical trial involving sleep apnea patients will be introduced to demonstrate the transferability and potential applications of our approach.

### 4.1 Performance Metrics

To evaluate the performance of the proposed approach, we employed two standard metrics commonly utilized in regression analyses: mean absolute error (MAE) and Pearson’s correlation coefficient (ρ). If $y_{i}\in Y$ denotes the from proposed approach estimated ${\mathrm{SpO}}_{2}$ and ${\hat{y}}_{i}\in \hat{Y}$ denotes their corresponding reference values, the MAE and ρ can be defined as

MAE(Y,Y^)=1n∑i=1n|yi−y^i|, (4)

ρ(Y,Y^)=∑i=1n(yi−Y¯)(y^i−Y^¯)∑i=1n(yi−Y¯)2∑i=1n(y^i−Y^¯)2. (5)

In addition, we introduced bias (B), also known as “mean,” to represent the average discrepancy between all estimated ${\mathrm{SpO}}_{2}$ values and their corresponding reference values. Meanwhile, the 95% limits of agreement (95% LoA) are defined as the range covering 1.96 times the standard deviation of these discrepancies, offering an insight into the consistency of our estimations.

### 4.2 Results with Proposed Approach

Table 2 summarizes the performance of ${\mathrm{SpO}}_{2}$ estimation with the proposed approach in the aforementioned two validation scenarios. Across both scenarios, the average correlation coefficients of all test measurements (Avg. ρ) remain robust, suggesting a strong correlation between estimated and actual ${\mathrm{SpO}}_{2}$ values. The bias (B) is generally low, indicating minimal systematic underestimation or overestimation. In the “Precision Healthcare” scenario, the overall MAE stands at 2.12%, with a slightly higher MAE of 2.41% observed during desaturation events. The “leave-one-participant-out” scenario exhibits an overall MAE of 2.31%, with desaturation events resulting in a higher MAE of 3.26%. No significant deterioration in results is noted across any specific skin type. However, skin type V displayed a notably better MAE compared to others, potentially due to the data obtained with narrow reference ${\mathrm{SpO}}_{2}$ values distribution from participants with this skin type.

**Table 2:** Results summary of the performance of the proposed approach.

Skin type	MAE (%)			Avg. ρ	B (%)	95% LoA (%)
	All	Normal	Desaturation			
**Precision healthcare scenario**						
II	2.06	1.63	2.45	0.72	−0.63	[−5.44, 4.17]
III	2.04	1.81	2.20	0.70	−0.13	[−5.59, 5.33]
IV	2.48	1.67	3.02	0.80	−1.16	[−7.77, 5.44]
V	1.38	0.84	1.83	0.55	−0.50	[−4.24, 3.23]
**All**	**2.12**	**1.71**	**2.41**	**0.72**	−**0.46**	**[−6.19, 5.27]**
**Leave-one-participant-out scenario**						
II	2.04	2.02	2.08	0.54	0.71	[−4.14, 5.55]
III	2.42	1.76	3.40	0.62	−0.51	[−6.76, 5.75]
IV	2.23	1.56	3.46	0.72	−0.05	[−6.15, 6.05]
V	1.80	1.98	1.28	0.68	1.31	[−2.33, 4.95]
**All**	**2.31**	**1.74**	**3.26**	**0.64**	−**0.20**	**[−6.29, 5.88]**

Considering the generalizability of the proposed approach and the prospect of practical applications, all the results presented and discussed next will be based on the more complex “leave-one-participant-out” scenario.

**Fig. 8:**
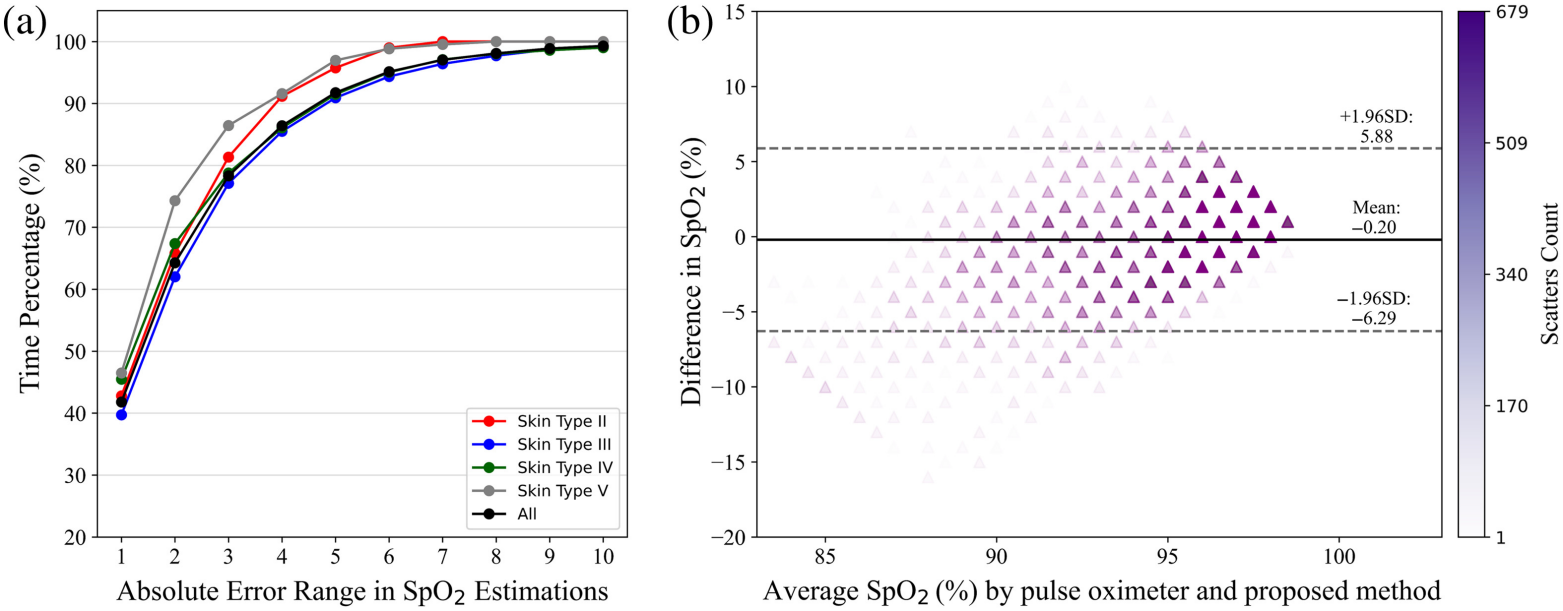
Performance visualization of the proposed approach in the “leave-one-participant-out” scenario. (a) The percentage of time (PERC) within the range of absolute error 1% to 10% between reference ${\mathrm{SpO}}_{2}$ and estimated ${\mathrm{SpO}}_{2}$. (b) The Bland–Altman plot shows the agreement between the proposed approach and the commercial pulse oximeter. The y-axis represents the differences between the estimated and reference ${\mathrm{SpO}}_{2}$, while the x-axis represents the average of the two values. Three lines represent respectively the mean difference (bias) and upper and lower 95% limit of agreement. The transparency of the triangle markers reflects the number of overlapping scatters.

**Fig. 9:**
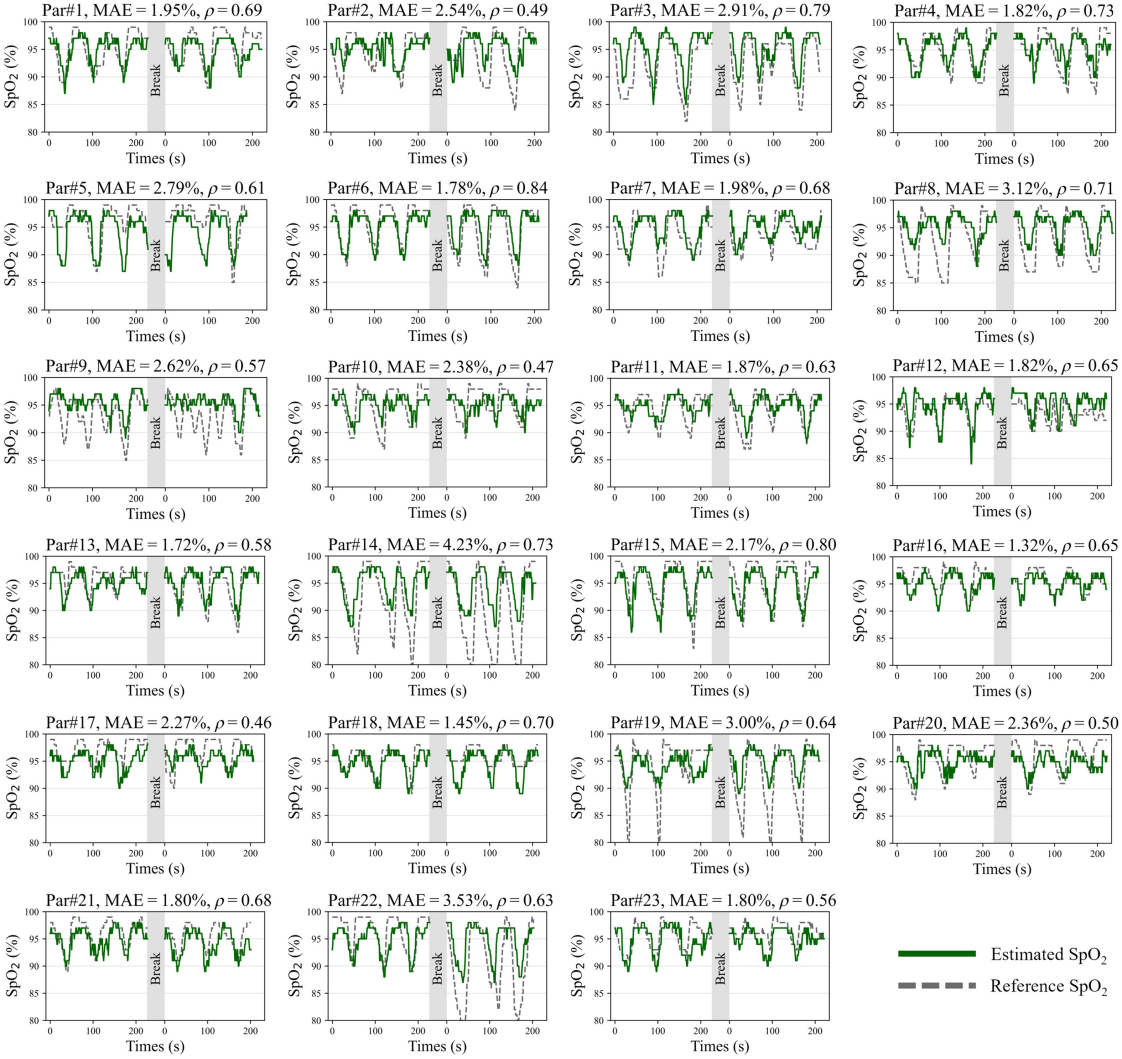
Estimated ${\mathrm{SpO}}_{2}$ values and pulse oximeter measured reference ${\mathrm{SpO}}_{2}$ values of 23 participants in the “leave-one-participant-out” scenario. For each participant, the model is trained by all measurements from the other 22 participants and tested on the left two measurements of this participant. Between two test measurements, there is a break. The green lines represent the estimated values, while the reference signals are dashed gray lines.

As shown in Fig. 8, we have introduced the percentage of time the discrepancy between the estimation and reference values falls within a certain range (PERC) and the Bland–Altman plot^52^ to analyze the agreement between our proposed approach and pulse oximeter recordings in the “leave-one-participant-out” scenario. It is observed in Fig. 8(a) that the discrepancy of estimated ${\mathrm{SpO}}_{2}$ values is within 3% of the reference values for ∼80% of all time points. Both Table 2 and Fig. 8(a) demonstrate that our approach does not perform significantly worse in estimating ${\mathrm{SpO}}_{2}$ for any specific skin type. Furthermore, as shown in Fig. 8(b), the vast majority of the data points in the Bland–Altman plot lie within the 95% LoA, suggesting a strong agreement between the two ${\mathrm{SpO}}_{2}$ measurement approaches. But the 95% LoA range from −6.29 to 5.88, which is higher compared to those reported in some classic works.^27^^,^^53^ This can be due to the wide distribution of ${\mathrm{SpO}}_{2}$ values in our dataset, which ranges from 80% to 99%, and includes a significant number of low-oxygen saturation values, with nearly 3% of values falling below 85%. Besides, the higher 95% LoA reflects the suboptimal performance of our approach in extreme cases, which may be due to the imbalance in the training data. Capturing more data with a low ${\mathrm{SpO}}_{2}$ level for supervised learning could be expected to improve this situation. The estimated ${\mathrm{SpO}}_{2}$ signals and reference signals in the “leave-one-participant-out” scenario for all the participants are presented in Fig. 9. We spliced two videos for one participant so that the ${\mathrm{SpO}}_{2}$ curves of each participant should contain several dips that result from breath-holding. The MAE of estimated and reference signals across the participants ranges from 1.57% to 3.53%, and the Pearson correlation coefficient varies from 0.49 to 0.73. For the majority of the time, even during the desaturation events, the estimated ${\mathrm{SpO}}_{2}$ values track closely with the reference ${\mathrm{SpO}}_{2}$ values, although some variations exist. As shown for Par#14, Par#19, and Par#22, when the reference ${\mathrm{SpO}}_{2}$ values are exceptionally low, typically below 85%, the estimated values indicate a downward trend but do not reach those low levels. This could be attributed to the scarcity of extremely low data points involved during the training.

### 4.3 Discussion of Feature Extraction Strategies and Network Structures

**Fig. 10:**
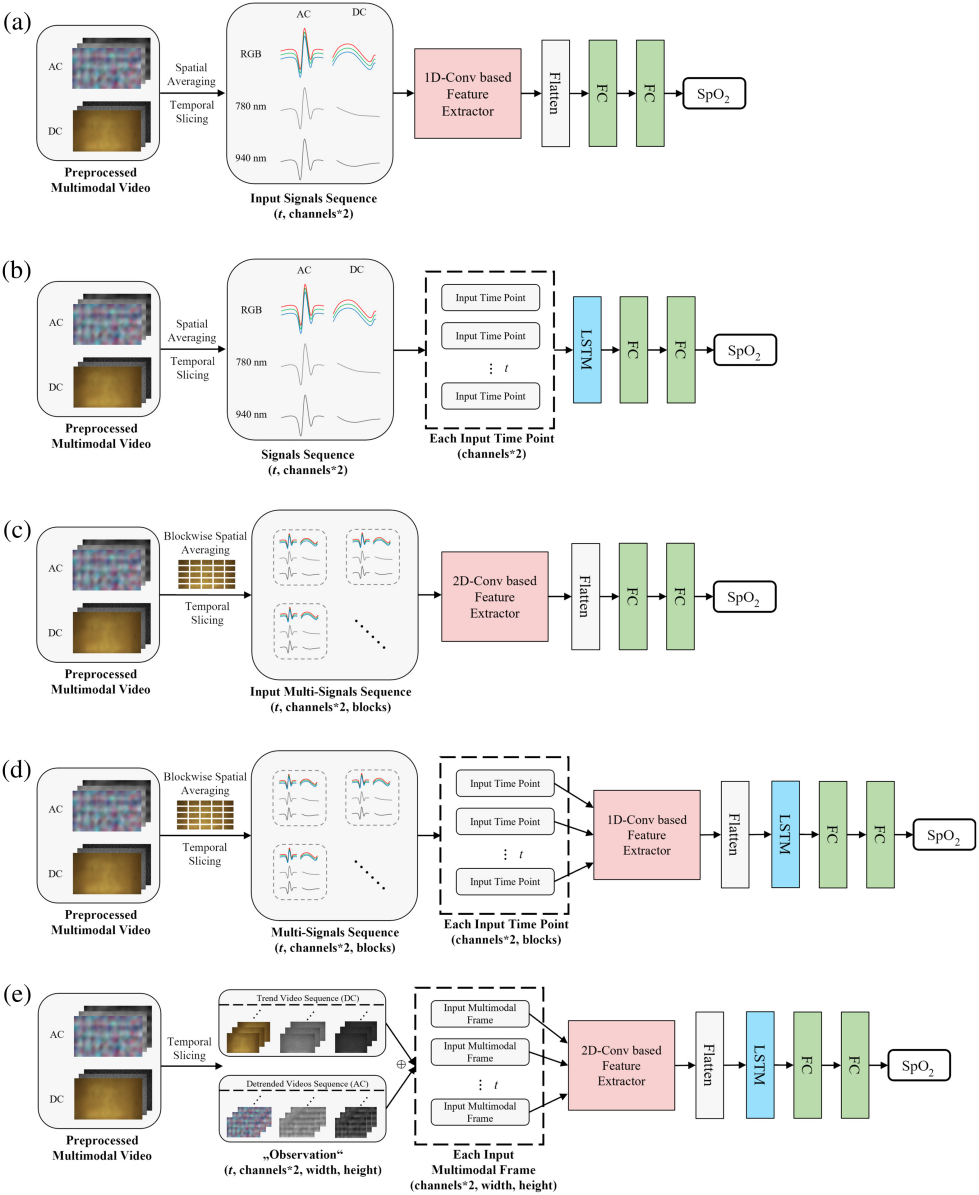
Schematic of different feature extraction strategies and corresponding network architectures. (a) Strategy A. (b) Strategy B. (c) Strategy C. (d) Strategy D. (e) Strategy E.

In our proposed approach, we treat a 1-s “observation,” namely a 15-frame video sequence of preprocessed multimodal videos with both DC and AC components, as the input to a 3D CNN feature extractor for simultaneous extraction of temporal and spatial features. In this subsection, we compare the performance of various feature extraction strategies and corresponding network architectures, which are schematically depicted in Fig. 10, with the proposed approach.

1. Strategy A: we process the DC and AC components of multimodal videos by averaging them spatially to obtain multimodal DC and AC signals. These signals are then sliced into 1-s sequences, each containing 15 time points, to serve as inputs for shallow 1D-CNN feature extractors. This strategy only focuses on temporal feature extraction but not spatial features.
2. Strategy B: similar to strategy A, this strategy begins by spatially averaging the DC and AC components of multimodal videos to obtain multimodal spatially averaged signal sequences. However, unlike strategy A, each time point within a sequence is flattened and fed into a long short-term memory (LSTM) network^54^ as a one-time step. The LSTM model outputs one ${\mathrm{SpO}}_{2}$ estimation value after processing all time points within the sequence. This strategy aims to capture the dependencies between different time points within the signal sequence. But there is also spatial feature neglect.
3. Strategy C: we blockwise spatial average the DC and AC components of multimodal videos and concatenate them to obtain multiple multimodal signals corresponding to the number of blocks. These multiple signals are then sliced into sequences as inputs for shallow 2D CNN feature extractor. In this way, both temporal features and some spatial features between the blocks are concurrently considered.
4. Strategy D: similar to strategy C, we can obtain multiple multimodal signals. Then, at each time point, multiple signal values from different channels are first processed through a 1D CNN to extract spatial features, which are then flattened and fed into an LSTM as a one-time step. The LSTM extract then temporal dependencies related features within the sequence.
5. Strategy E: each frame of an “observation” is processed through a 2D CNN feature extractor to get spatial features. Subsequently, the features of each frame are flattened and serve as a one-time step for the LSTM. This approach initially extracts features in the spatial domain and then analyzes temporal dependencies within these spatial features.

**Fig. 11:**
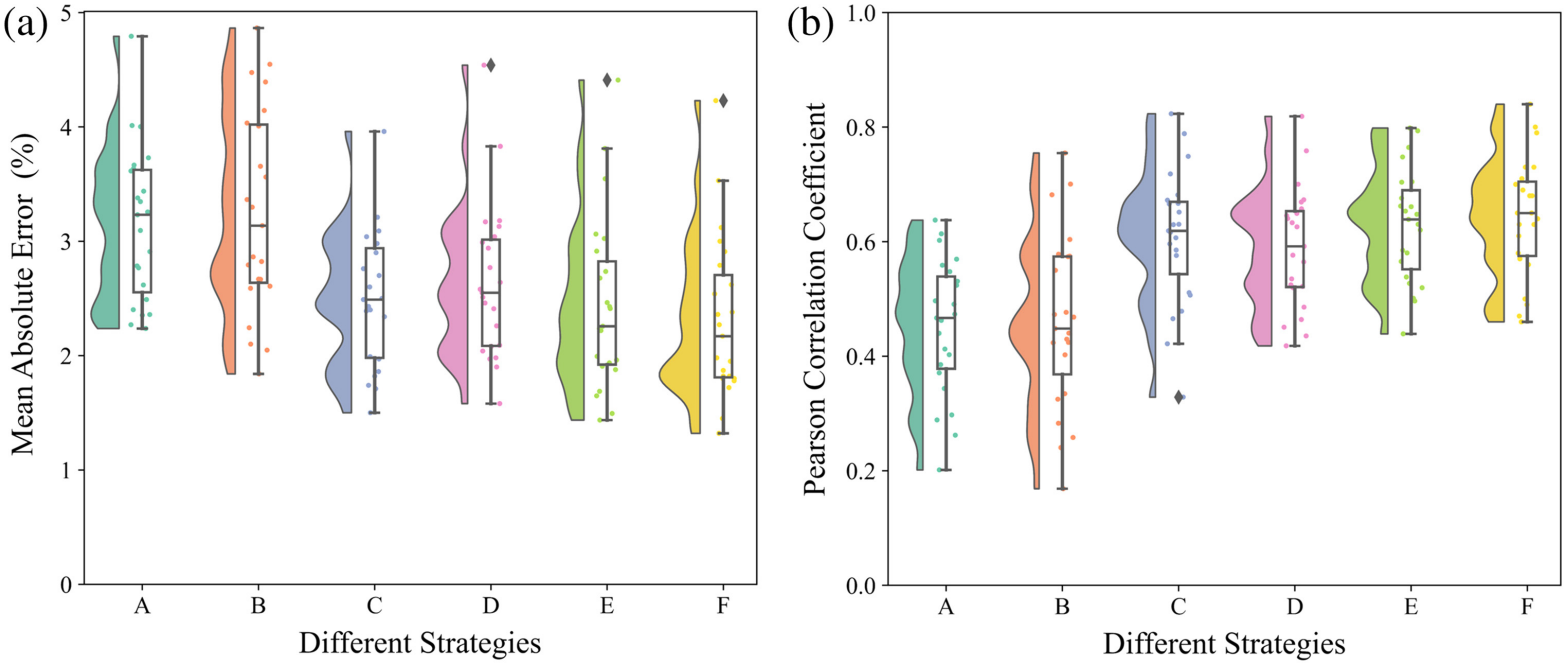
Raincloud plots, which combine elements of box plots, violin plots (“cloud” part), and scatter plots (“rain” part), for performance metrics across different feature extraction strategies. The “cloud” part represents result distribution, while the “rain” indicates individual results of 23 participants. For each metric, boxes are used to describe the interquartile range (IQR) of the “leave-one-participant-out” test results on 23 participants with different strategies, which spans from the 25th percentile (Q1) to the 75th percentile (Q3). The whiskers extending from the boxes represent non-outlier results within 1.5 times IQR. The lines inside the boxes are medians. (a) Mean absolute error (MAE). (b) Pearson correlation coefficient (ρ).

As shown in Table 3, the proposed strategy (strategy F), in which temporal and spatial features are simultaneously extracted by 3D CNN, yields the best regression outputs. The distributions of MAE and Pearson correlation coefficients for the estimation ${\mathrm{SpO}}_{2}$ using different strategies compared to reference values across 23 participants are presented in Fig. 11. It is noteworthy that in terms of both MAE and Pearson correlation coefficients, the proposed strategy demonstrates better results statistics (median, Q1, Q3) and distribution. Besides, strategies C and E exhibited similar performances and both achieved an MAE below 2.5 and an average Pearson correlation coefficient above 0.6.

**Table 3:** Result comparison between different feature extraction strategies.

Index	Feature extraction strategy	Network as feature extractor	MAE (%)	Avg. ρ
A	Only temporal features	1D CNN	3.14	0.45
B	Only temporal features	LSTM	3.24	0.46
C	Simultaneously extract temporal features and spatial features between blocks	2D CNN	2.49	0.61
D	Extract firstly spatial features between blocks and then temporal features	1D CNN + LSTM	2.63	0.59
E	Extract firstly spatial features on each frame and then temporal features	2D CNN + LSTM	2.43	0.63
**F (proposed)**	**Simultaneously extract spatial** **and temporal features**	**3D CNN (ResNet 18)**	**2.31**	**0.64**

We also compared different 3D CNN structures for feature extraction, considering both regression performance and the complexity of the models (size and computational load). Therefore, MACs, inference time, and the number of learnable parameters are introduced for a comprehensive evaluation of the network structure’s performance. As shown in Table 4, 3D ResNet 10, 3D ResNet 18, and 3D ResNet 34 have no significant difference in regression performance for our task, while 3D AlexNet performs comparatively worse. Our proposed approach is just to choose the best network structure based on MAE and Avg. ρ, which is 3D ResNet 18. Lightweight networks such as 3D MobileNet V1, 3D MobileNet V2, 3D ShuffleNet V1, and 3D ShuffleNet V2 significantly reduce model complexity without a noticeable loss in regression performance.^55^ Among them, 3D ShuffleNet V2 performs the best in these lightweight networks, achieving 2.57% MAE and 0.59 average Pearson correlation coefficient, which provides valuable reference for potential applications on mobile and embedded platforms.

**Table 4:** Performance comparison between different 3D CNN-based networks as feature extractor.

Model	MACs (G)	Inference Time (ms)	Learnable Parameters (M)	MAE (%)	Avg. ρ
3D AlexNet	0.73	28.10	2.07	2.81	0.52
3D ResNet 10	3.49	34.90	14.56	2.33	0.64
3D ResNet 18	4.27	45.80	33.36	2.31	0.64
3D ResNet 34	5.55	69.10	63.67	2.36	0.63
3D MobileNet V1	0.14	29.90	3.31	2.61	0.59
3D MobileNet V2	0.22	44.40	2.36	2.73	0.57
3D ShuffleNet V1	0.11	52.30	0.95	2.87	0.52
3D ShuffleNet V2	0.11	30.90	1.30	2.57	0.59

### 4.4 Discussion of Image Modalities

**Fig. 12:**
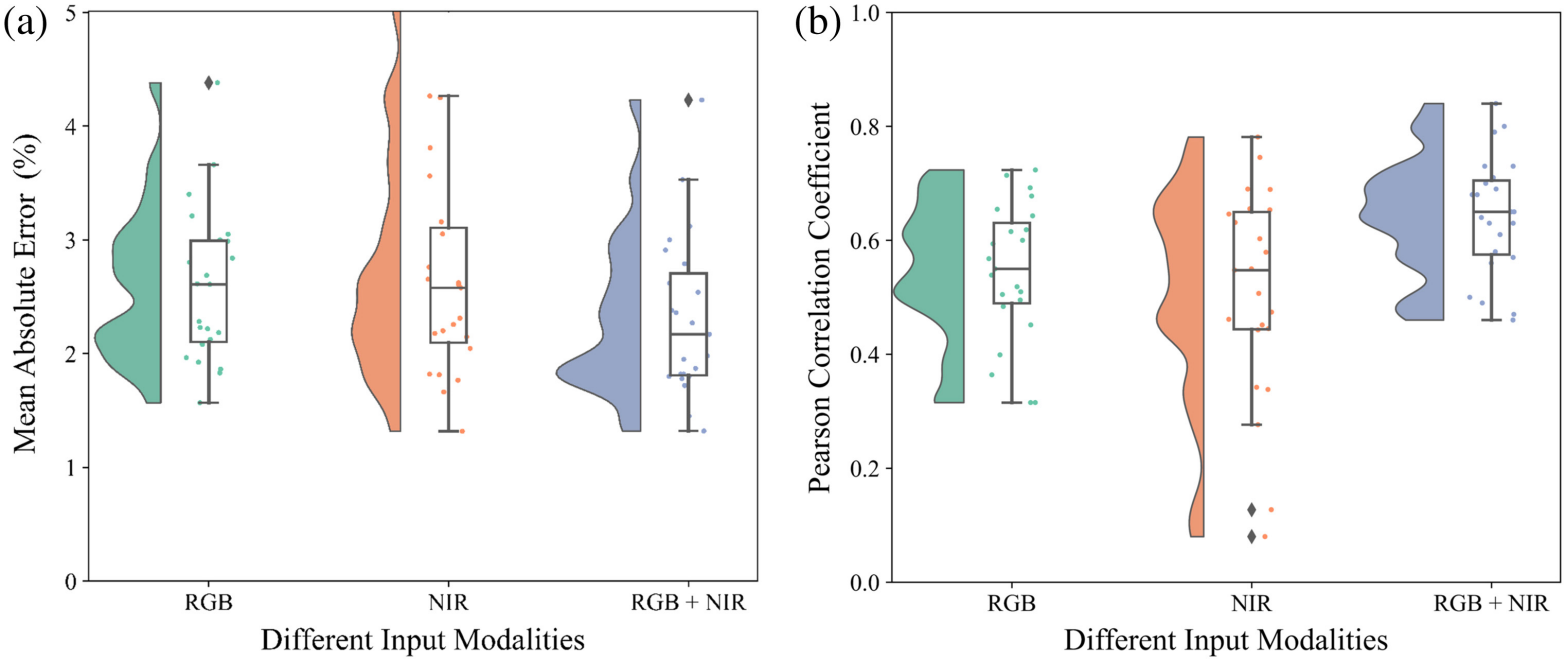
Raincloud plots, which combine elements of box plots, violin plots (“cloud” part), and scatter plots (“rain” part), for performance metrics across different input modalities. The “cloud” part represents result distribution, while the “rain” indicates individual results of 23 participants. For each metric, boxes are used to describe the interquartile range (IQR) of the “leave-one-participant-out” test results on 23 participants with different strategies, which spans from the 25th percentile (Q1) to the 75th percentile (Q3). The whiskers extending from the boxes represent non-outlier results within 1.5 times IQR. The lines inside the boxes are medians. (a) Mean absolute error (MAE). (b) Pearson correlation coefficient (ρ).

After image registration, our method permits the combination of different imaging modalities for ${\mathrm{SpO}}_{2}$ regression. Utilizing only NIR 780 and NIR 940 nm allows for overnight measurement, thereby broadening the applicability of this approach, such as in sleep monitoring scenarios. Table 5 illustrates the overall results when employing different modalities. Although the concurrent use of both RGB and NIR modalities yields the best estimation performance, relying solely on RGB or NIR does not lead to a collapse but only a slight MAE increase and an acceptable decrease of the Pearson correlation coefficient. From Fig. 12, it can be seen that estimations using only NIR resulted in a slightly higher MAE distribution for several participants and presented completely outlying Pearson correlation coefficients for two participants. However, in general, the distribution of the estimation results is similar to that when only RGB is used.

**Table 5:** Comparison of different input 2D imaging modalities.

Input modalities	Overnight measurement	MAE (%)	Avg. ρ
RGB	-	2.58	0.55
NIR	✓	2.68	0.51
RGB + NIR	-	2.31	0.64

### 4.5 Clinical Validation on Sleep Apnea Patients

To clinically validate our method, we conducted a patient study in cooperation with the Center for Sleep and Telemedicine, University Medicine Essen, and recruited four patients with suspected SAS. SAS is a sleep-related breathing disorder characterized by repetitive breathing interruptions during sleep, resulting in daytime drowsiness, concentration difficulties, and increased risk of cardiovascular diseases. Furthermore, recurrent breathing interruptions lead to a decrease in blood oxygen levels and eventually hypoxemia. The age of the included patients ranged from 51 to 58, while their apnea-hypopnea index (AHI) ranged from 29 to 69.9 and the oxygen desaturation index (ODI) from 10.7 to 62.8. AHI measures the severity of sleep apnea by calculating the number of apnea and hypopnea events per hour of sleep, while ODI quantifies the frequency of oxygen desaturation events, specifically drops of 3% or more, per hour of sleep.^56^^,^^57^ Each patient is assigned a unique identifier, ranging from patients #1 to #4. The study is approved by the Faculty of Medicine, University of Duisburg-Essen (approval no. 21-10312-BO). Informed consent is obtained from all individual patients. These patients spent one night in the sleep laboratory, being simultaneously monitored by our camera system and the PSG system for reference. The color camera of our system is inactive during the measurement. Thus, the previously RGB-based facial landmark extraction and forehead ROI definition have been shifted to operate on NIR 780 images. In this experimental phase, our camera system’s sensor head cannot move or rotate, resulting in a fixed field of view. We can check that the patient’s face is within the camera’s view at the start of recording, but patients might turn or move their heads after falling asleep. Therefore, in Table 6, we list some information about these four patients with their total sleep hours, the corresponding duration of available data, and MAE between estimated ${\mathrm{SpO}}_{2}$ and reference in this duration.

**Table 6:** Results of ${\mathrm{SpO}}_{2}$ estimation in trial clinical validation on SAS patients.

	Age (years)	AHI	ODI	Sleep time (h)	Available data (h)	MAE (%)
Patient #1	51	29.0	34.6	6.23	3.61	2.17
Patient #2	56	69.9	78.0	5.62	4.76	1.97
Patient #3	80	20.9	10.7	5.40	3.69	1.34
Patient #4	58	59.7	62.8	6.18	1.16	1.19

**Fig. 13:**
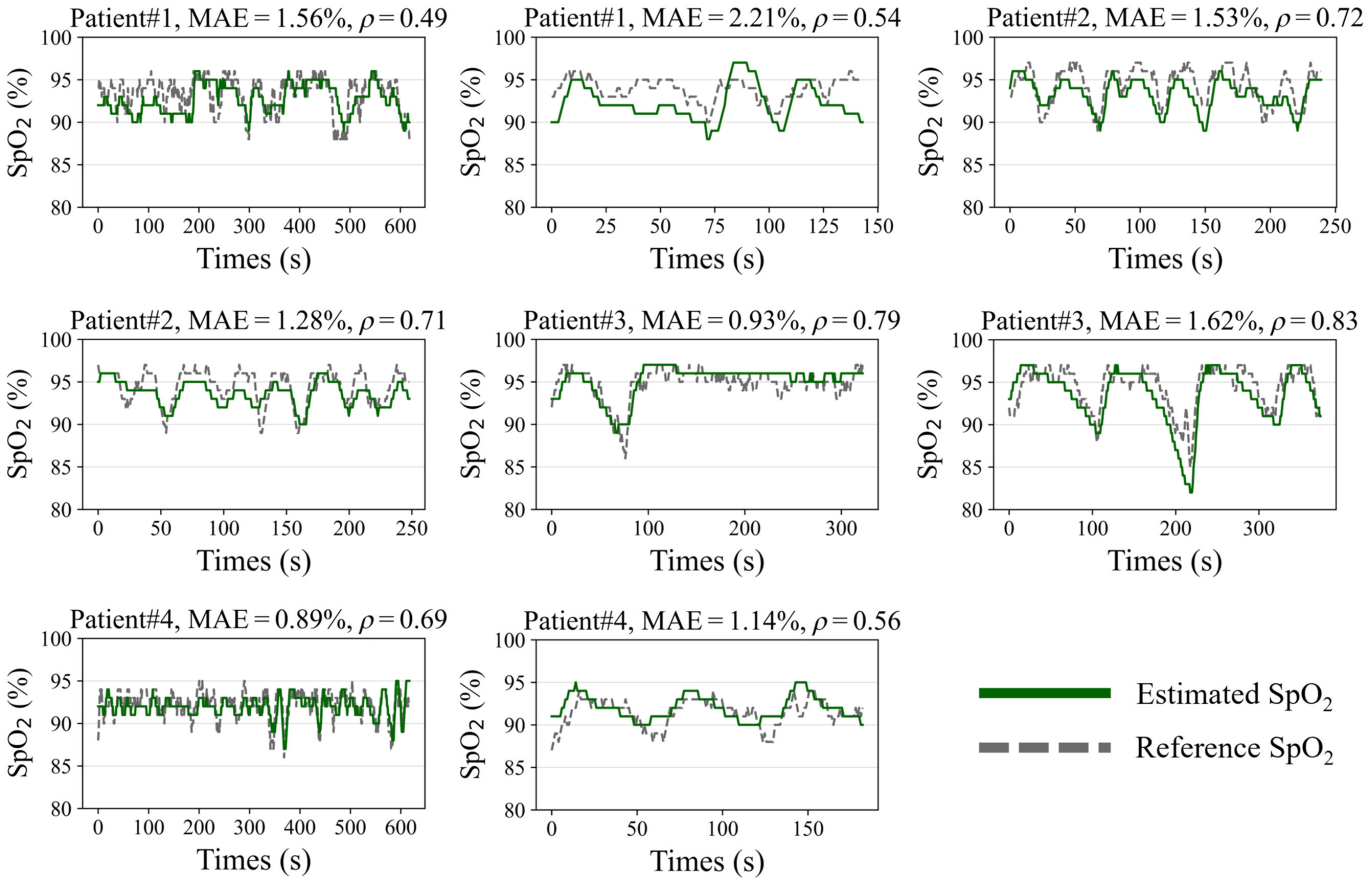
Estimated ${\mathrm{SpO}}_{2}$ values and PSG measured reference ${\mathrm{SpO}}_{2}$ values of 4 SAS patients (two periods with desaturation events for each patient). The model is trained by all measurements from 23 healthy participants. The test was conducted at night and used only the infrared channels. The green lines represent the estimated values, while the reference signals are dashed gray lines.

To provide a more intuitive demonstration of the clinical results, we demonstrate in Fig. 13 the dynamic response of the estimated and reference ${\mathrm{SpO}}_{2}$ signals during periods with desaturation and resaturation events. For each patient, two separate time periods with desaturation events are presented in two consecutive subplots. In a previous article of our research group,^58^ we showed that we can distinguish periods with and without desaturation events in SAS patients, however without estimating the ${\mathrm{SpO}}_{2}$ value. In this study, we show that we are able to accurately estimate the ${\mathrm{SpO}}_{2}$ value in patients with a highly dynamic ${\mathrm{SpO}}_{2}$ behavior with low MAE and high Pearson correlation coefficient. Furthermore, we have shown that the approach developed on healthy awake subjects can be applied to symptomatic SAS patients during sleep.

## 5 Conclusion and Future Work

This study introduced a contactless approach for ${\mathrm{SpO}}_{2}$ estimation using 3D CNN and 3D VIS-NIR multimodal imaging. Through multimodal image registration, accurate 3D ROI tracking, multimodal video preprocessing, and spatial-temporal feature extraction, oxygen saturation can be accurately estimated from facial videos. The approach exhibited promising results, achieving an MAE of 2.31% and a Pearson correlation coefficient of 0.64 in a breath-holding study on healthy participants during short-term daytime measurements, showing a strong response to desaturation events and good agreement with recordings from contact-based commercial pulse oximeters. In clinical trials involving patients with sleep apnea syndrome, our approach demonstrated robust performance, with an MAE of less than 2% in ${\mathrm{SpO}}_{2}$ estimations compared to gold-standard polysomnography (PSG). For the further improvement of ${\mathrm{SpO}}_{2}$ estimation, we plan to utilize 3D information to incorporate illumination correction, aiming to further reduce distortions that are unrelated to oxygen saturation. Besides, future studies will focus on expanding the dataset to include a broader range of real patients, including varied skin types and more extensive pathological conditions (both stationary and ambulatory settings), to further validate the approach’s effectiveness and generalizability. Furthermore, we aim to combine other non-contact measured vital signs, such as heart rate, respiration, and oxygen saturation, for correlation analysis to enhance disease diagnosis and patient recovery process monitoring.

## Data Availability

Due to privacy or ethical restrictions, the raw data used in this article is not publicly available but can be made available by the authors upon reasonable request.
